# Efficiency of an inexpensive liquid-based cytology performed by cytocentrifugations: a comparative study using the histology as reference standard

**DOI:** 10.1186/1742-6413-2-15

**Published:** 2005-09-15

**Authors:** Christian Garbar, Corinne Mascaux, Véronique Fontaine

**Affiliations:** 1 Department of Pathology, CHU de Charleroi (Université Libre de Bruxelles), 1 Boulevard Zoé Drion, 6000 Charleroi, Belgium; 2 Laboratory of Molecular Virology, Pasteur Institute, 642 rue Engeland, 1180 Brussels, Belgium

## Abstract

**Background:**

Although liquid-based cytology (LBC) is now recommended for cervical cancer screening, it requires expensive automated devices and materials. To evaluate the efficiency of inexpensive LBC methods relying on an inexpensive fixative liquid, Easyfix^®^, we compared the results obtained by the liquid-based cytology (LBC) diagnoses performed by cytocentrifugations (Papspin^® ^and Turbitec^®^) with those obtained by histology. Furthermore, we evaluated the efficiency of the fixative liquid, Easyfix^®^, to preserve HPV DNA in the collected samples.

**Method:**

266 LBC were compared with 174 colposcopies and 91 Loop Electrosurgical Excision Procedure (LEEP). Among the LBC, 51 were performed using the Papspin^® ^system and 215 were performed using the Turbitec^® ^system. To control the quality of the preservation liquid, Easyfix^®^, we correlated the results of HCII assays with those of HPV PCR.

**Results:**

For Papspin^® ^and Turbitec^® ^systems, the sensitivities were respectively 82.6% (95% CI: 61.2–95.0%, p < 0.001) and 75.0% (95% CI: 64.4–89.8%, p < 0.001) and the specificities were 92.6% (95%CI: 76.5–99.1%, p < 0.001) and 96.2% (95% CI: 91.3–98.7%, p < 0.001). We find no statistical difference between the results of the both systems (p = ns). The sensitivity of the HCII was 86.4% (95% IC: 77.4–92.8%, p < 0.001) and the specificity was 39.4% (95% CI: 31.2–48.1%, p < 0.001). The comparison between HCII and HPV-PCR shows a good correlation: the kappa was 0.89.

**Conclusion:**

LBC performed by cytocentrifugations are inexpensive, reduce inadequate smears, show excellent efficiency and allow HPV detection by molecular biology.

## Background

For more than ten years, liquid-based cytology (LBC) has been developed for cervical cancer screening. Unlike to Conventional cervical Smears (CS), cells are scattered in a fixative liquid to produce a thin layer of cells on slides. The main advantages of this technique are to reduce the number of inadequate smears and to provide enough cells for the detection of infectious agents such as human papillomavirus (HPV) through molecular biology techniques [1-3]. At the moment, the majority of these techniques are using expensive automated devices leading to a significant increase in the price of LBC [4,5].

The LBC performed by cytocentrifugations exist since the 1970s and have been developed for the automated reading of cervical cancer cytology. Preliminary studies show that they significantly reduce the cost of LBC [6-13]. In Central Europe, the LBC performed by cytocentrifugation consist mainly in the Papspin^® ^system (ThermoShandon Inc, Pittsburgh, the USA), in the CytoSCREEN^® ^system (Seroa, Monaco, Monaco) and in the Turbitec^® ^system (Labonord, Templemars, France). These last two techniques use the Hettich cytocentrifuge (Andreas Hettich Corp, Tuttligen, Germany).

The first purpose of this study was to evaluate the performance of two LBC performed by centrifugations: the Papsin^® ^and the Turbitec^® ^systems. In addition, we evaluated the efficiency of the fixative liquid, Easyfix^® ^(Labonord, Templemars, France) to preserve HPV DNA.

## Materials and methods

### Patients

A total of 268 LBC were collected from 177 women (mean of age: 37.3 +/-10.8 years) from January 2002 to December 2004 in a routine gynecologic setting at the Department of Gynecology (CHU de Charleroi, Belgium). Indications for gynecological consultation were based on previous abnormal conventional cervical smears in a population-based screening. According to the histological diagnosis, the age groups were quite homogeneous, except for the women in the CIN3 group who were slightly younger than those of the other groups (Table 1, p = 0.04).

**Table 1:** Patients age distribution and samples distribution among the Papspin^® ^and Turbitec^® ^LBC according to the histological diagnosis.

**Histology diagnosis**	**Papspin^®^**	**Age **(Years)*	**Turbitec^®^**	**Age **(Years)*
**WNL**	**12/51 **(23.5 %)	**45.1 +/- 15.6**	**86/215 **(39.9%)	**38.3 +/- 11.9**
**CIN 1**	**16/51 **(31.3%)	**42.6 +/- 8.9**	**46/215 **(21.1%)	**37.3 +/- 11,6**
**CIN 2**	**4/51 **(7.8%)	**38.6 +/- 2.8**	**15/215 **(6.9%)	**39.6 +/- 13.2**
**CIN 3**	**19/51 **(37.2%)	**33.5 +/- 7.9**	**70/215 **(32.2%)	**34.3 +/- 7.8**

174 colposcopies associated with biopsy (30 for Papspin^® ^and 144 for Turbitec^®^) and 92 Loop Electrosurgical Excision Procedure (LEEP) (21 for Papspin^® ^and 71 for Turbitec^®^) were correlated with all LBC. All the cells were collected using a Cervexbrush^® ^(Rovers, Oss, Nederlands), immersed in 15 ml of a non-buffered alcoholic fixative liquid containing 30% ethanol (Easyfix^®^, Labonord Corp, Templemars, France). 2 LBC corresponding to a cytological and histological diagnosis of cervical adenocarcinoma were discarded. All the patients were informed of the purpose of this study and agreed voluntarily to take part in it.

### Methods

The Easyfix^® ^cell fixative solutions, containing the Cervexbrush^® ^and the cells, were homogenized by mechanical agitation (Vortex^®^) for at least 30 seconds.

For the *Turbitec*^® ^*technique*, 4 drops albumin (StickOn^®^, Labonord) were put on a polylysined slide placed in the centrifuge chamber (Hettich Centrifuge^®^). The centrifuge chambers had a final volume of 6 ml; the surface of cells projection on the slide was 240 mm^2^. The cellularity was estimated using a photoelectric cell analyzer (Labonord) according to the dilution of the Easyfix^® ^sample: 200 μl, 500 μl, 1 ml, 3 ml or 5 ml of Easyfix^® ^solution. This volume was placed in the centrifuge chamber and diluted with an alcoholic fixative liquid containing polyethylene glycol (Cytofix^®^, Labonord), in order to obtain a finale volume of 6 ml. After 10 minutes centrifugation at 2000 revolutions per minute (rpm), the liquid was discarded.

The *Papspin*^® ^*technique *was performed as described previously in the literature with the exception of the use of the Easyfix^® ^and the Cytofix^® ^fixative liquids [14]. The surface of cells projection was 300 mm^2^. The volume of dilution was respectively 500 μl, 1 ml and 2 ml and the finale volume was 3 ml, according to the turbidity of the sample. The chambers (Megafunnel^® ^– ThermoShandon) were centrifuged 5 minutes at 1250 rpm (CytoSpin^® ^– ThermoShandon) [9-12].

For cytology diagnosis, we used the Bethesda system 2001: within the normal limits (WNL), atypical squamous cells cannot exclude high grade squamous intraepithelial lesion (ASC-H), atypical squamous cells of undetermined significance (ASC-US), low and high grade squamous intraepithelial lesions (Lg-SIL and Hg-SIL) [14]. The histology diagnoses were classified in 4 groups: WNL, Cervical Intraepithelial Neoplasia grade 1 (CIN 1), grade 2 (CIN 2) and grade 3 (CIN3). For the cytology diagnosis and the HCII results, the reference standard was the CIN2 and above (CIN2+). No cytology or histology diagnoses were revised.

225 HPV tests performed by the *Hybrid Capture II*^® ^(Digene Corp, Beltsville/HCII) were also realized (38 for Papspin^® ^and 187 for Turbitec^®^). The HCII assays were performed on 5 ml of the residual liquid-based samples. After 5 min. centrifugation at 2000 rpm, the supernatant was discarded. The cellular pellet was washed once with 1 ml PBS, resuspended in 100 μl of Cervical Sample ^® ^(Digene Corp, Beltsville) and denatured in an alkaline solution. Classical hybridization, detection and calibration were made according to the HCII kit's instructions [15,16]. We only used the probes against the high-risk HPV: 16, 18, 31, 33, 35, 39, 45, 51, 52, 56, 58, 59, and 68. The HCII results were expressed as positive or as negative depending on the relative light unit of 1 pg/ml of HPV DNA.

We also performed PCR techniques to detect HPV DNA in 72 samples fixed in Easyfix^® ^solutions for 3.2 +/- 0.9 months. Total cellular DNA were extracted by a freeze/defreeze method as previously described [17] or were purified using the QIAamp blood minikit (Qiagen). The quality of the extracted DNA was evaluated by PCR using beta-globin specific primers, as described [18]. HPV-specific PCR were performed using the general primers, as previously described [19].

### Statistics

A Chi ^2 ^or a Fisher exact, ANOVA and kappa of Cohen tests were performed. A p-value less than 0.05 was considered statistically significant. The results are expressed as a mean +/- standard deviation or as a percentage.

We use the Epi Info 2002 revised 2003 (Centers of Disease Control and Prevention – W.H.O, Atlanta, USA) and the Analyse-it^® ^1.7 (Analyse-it Software, Leeds, England) programs.

## Results

**Figure 1:**
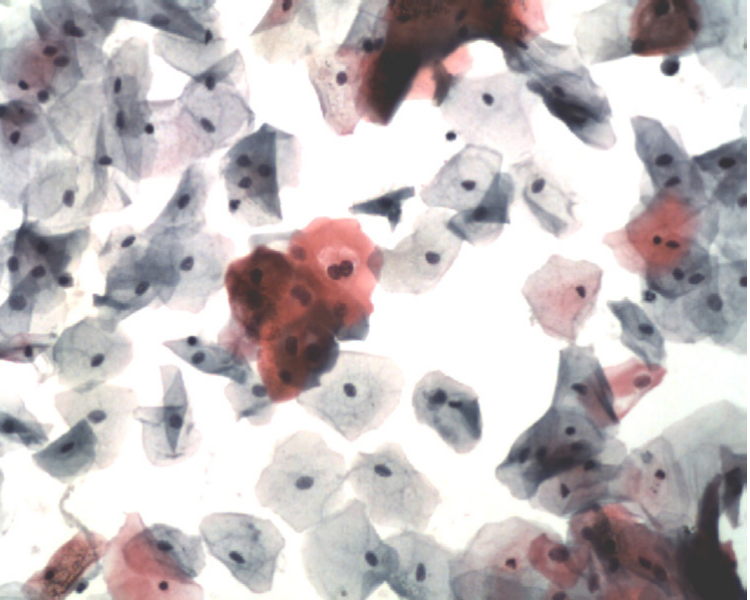
Lg-SIL : cellularity is satisfactory (Papanicolaou stain, high magnification – 20x objective, Papspin^® ^system). HCII was positive.

**Figure 2:**
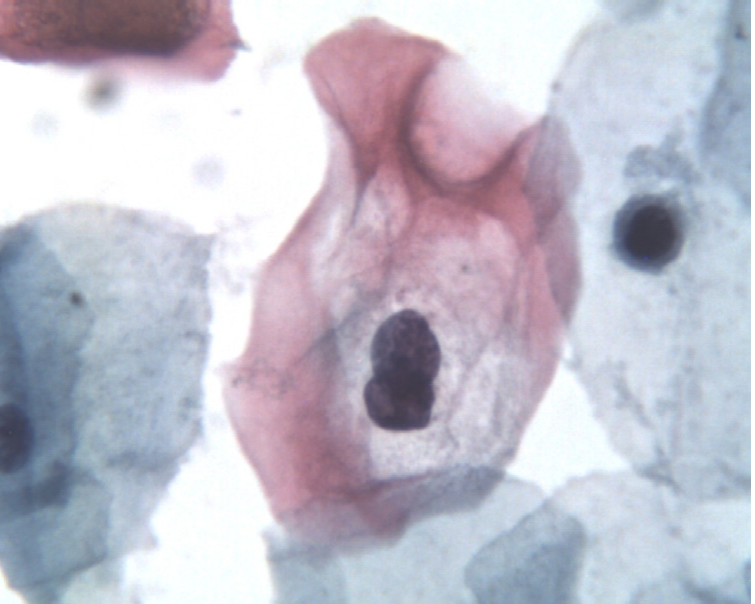
Same case a high magnification : see cytoplasmic and nuclear details (Papanicolaou stain, high magnification – 100x objective, Papspin^® ^system).

**Figure 3:**
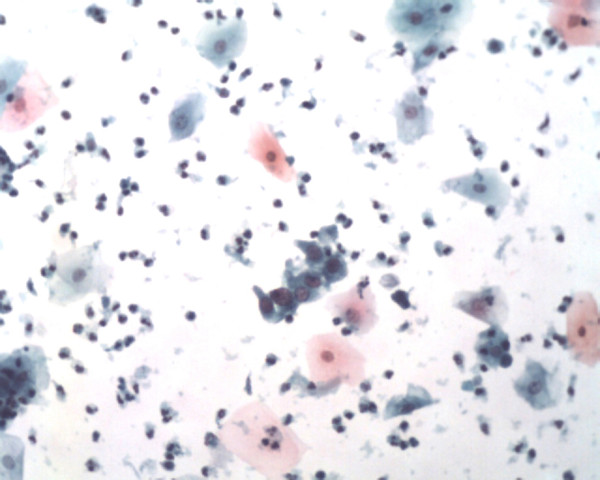
Hg-SIL : presence of inflammatory cells which did not influenced the lecture (Papanicolaou stain, high magnification – 20x objective, Turbitec^® ^system). HCII was positive.

**Figure 4:**
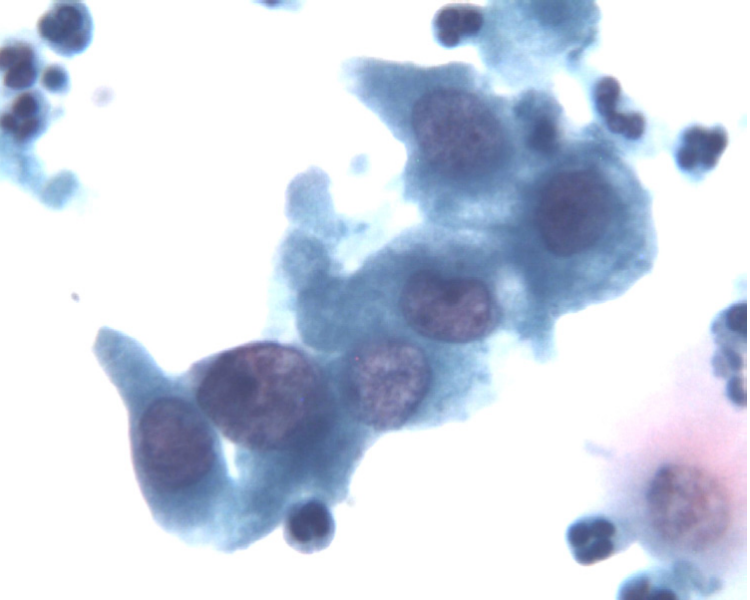
Same picture a high magnification : see cytoplasmic and nuclear details (Papanicolaou stain, high magnification – 40x objective, Turbitec^® ^system).

The microscopic reading of the LBC, performed by cytocentrifugations, was virtually the same as for other classical LBC techniques, especially the Prepstain^® ^system, although a higher background (cell debris, inflammatory cells, lactobacillus, blood...) was observed but did not influenced the lecture (figures 1 to 4).

According to Bethesda 2001, only 2 smears (0.9%) were classified as inadequate for cytology interpretation because of too low cellularity. These two smears were performed by Turbitec^®^. Indicative minor quality criteria were absence of cells junction for 11/51 Papspin^® ^(21.5%) vs 24/215 for Turbitec^® ^(11.1%), low cellularity for 1/51 (1.9%) vs 4/215 (1.8%), hemorrhage for 2/51 (3.8%) vs 6/215 (2.7%), severe inflammation for 1/51 (1.9%) vs 1/215 (0.4%) and severe cytolysis for 2/51 (3.8%) vs 2/215 (0.9%) of LBC. However, we can not draw any statistical conclusion because of the too low number of LBC.

In order to evaluate the performance of the LBC performed by cytocentrifugations from ours clinical samples preserved in the Easyfix^® ^liquid, we compared the results of 2 methods of LBC centrifugations with the histological diagnosis.

According to the histology diagnosis, the samples were homogeneously distributed between the Turbitec^® ^and the Papspin^® ^cytocentrifugation systems (Table 1). There was a slight significant difference between the distribution of the histology diagnosis of the Turbitec^® ^and Papspin^® ^systems only for the group of the WNL (Table 1, p = 0,02).

The comparison between the results obtained by the two cytocentrifugation LBC and those obtained by histology is shown in Table 2. There was no statistical difference with caution of the small number of Papspin^® ^sample. The global tendency was very similar except for ASC-H which are more frequently cited for Turbitec^® ^(1/51 or 1.9% vs 13/215 or 6.0%, p = ns). This is probably due to the fact that Papspin^® ^LBC were performed after Turbitec^® ^LBC when ours cytologists and pathologists were more used to the Bethesda system 2001.

**Table 2:** Comparison of cytological and histological diagnosis of Papspin^® ^and Turbitec^®^.

Histological diagnosis	Cytological Diagnosis **Papspin^®^**				
	**WNL**	**ASC-US**	**ASC-H**	**Lg-SIL**	**Hg-SIL**
**WNL**	**5/6 **(83.3%)	**2/5 **(40.0%)	**-**	**4/18 **(27.8%)	**-**
**CIN 1**	**1/6 **(16.7%)	**3/5 **(60.0%)	**1/1 **(100%)	**9/18 **(50.0%)	**2/21 **(9.5%)
**CIN 2**	**-**	**-**	**-**	**2/18 **(5.5%)	**3/21 **(14.3%)
**CIN 3**	**-**	**-**	**-**	**3/18 **(16.7%)	**16/21 **(76.2%)

	**Turbitec^®^**				
**WNL**	**37/42 **(88.1%)	**17/29 **(58.6%)	**6/13 **(46.1%)	**22/63 **(34.9%)	**2/68 **(2.9%)
**CIN 1**	**5/42 **(11.9%)	**7/29 **(24.1%)	**1/13 **(7.8%)	**30/63 **(47.6%)	**3/68 **(4.4%)
**CIN 2**	**-**	**-**	**-**	**2/63 **(3.3%)	**13/68 **(19.1%)
**CIN 3**	**-**	**5/29 **(17.3%)	**6/13 **(46.1%)	**9/63 **(14.3%)	**50/68 **(73.6%)

38 samples from the Papspin^® ^and 187 from the Turbitec^® ^methods were also analyzed in the HCII assay. The frequency of HPV performed by HCII was respectively for Papspin^® ^and Turbitec^® ^of 50.0% vs 30.0% for WNL, 60.0% and 51.8% for ASC-US, 100% vs 100% for ASC-H, 87.5% vs 71.4% for Lg-SIL and 100% vs 85.0% for Hg-SIL. Those results suggested that HPV DNA was more detected among the ASC-US than among the WNL and that the HPV DNA is more frequently detected for ASC-H, Lg-SIL and Hg-SIL. However, because of the small cases in each group, no conclusion can be made.

The sensitivity and the specificity of Papspin^® ^and Turbitec^® ^methods were calculated using the CIN2+ histology detection as the reference standard for the cytological diagnosis of Hg-SIL. Respectively for Papspin^® ^and Turbitec^®^, the sensitivity was 82.6% (95%CI: 61.2–95.0%, p < 0.001) and 75.0% (95% CI: 64.4–89.8%, p < 0.001) and the specificity was 92.6% (95%CI: 76.5–99.1%, p < 0.001) and 96.2% (95%CI: 91.3–97.7%, p < 0.001). There was no statistical difference between the 2 LBC systems (p = ns).

The false negatives LBC (with CIN2+ as reference standard) consist of 4 Papspin^®^, all Lg-SIL and all positive for HPV/HCII (4/4) and of 22 Turbitec^®^, 11 Lg-SIL (8/11 positive for HCII), 5 ASC-US (4/5 positive for HCII) and 6 ASC-H (6/6 positive for HCII). The false positives LBC consist of 2 for Papspin^® ^and 4 for Turbitec^®^, all positive for HCII (2/2 and 4/4) and all with a colposcopy with only one small biopsy which is no representative of the whole cervix.

We also evaluated the quality of the Easyfix^® ^fixative liquid to preserve HPV DNA in the residual materials of 72 LBC performed by cytocentrifugations. Even after 3.2+/-0.9 months, at room temperature, beta-globin or HPV DNA could still be amplified by PCR in those samples. The comparison between HPV detection using either general primer PCR or HCII tests showed a Kappa test of 0.89 (p < 0.001). Three cases were positive for the HPV PCR and negative for the HCII and one case was negative for the HPV PCR and positive for the HCII. This last case was also negative for the beta-globin PCR explaining that HPV could not be detected because of the poor quality of the extracted cellular DNA. The stability of DNA in the Easyfix^® ^medium after 3 months was in general excellent.

## Discussion

In this study, we demonstrated the feasibility and the efficiency of inexpensive LBC performed by cytocentrifugations: the Papspin^® ^and the Turbitec^® ^systems. We use colposcopy with biopsy or LEEP as reference standard. The diagnosis threshold was set up to the high-grade cervical intraepithelial neoplasia (CIN2+). This could have led to a selection bias of a high-risk patient population. Therefore, ours results are only acceptable in the framework of diagnosis but must be interpreted with some caution in the framework of cervical cancer screening in which the number of healthy patients should usually be as high as possible.

The adequacy of the LBC has been already described in the literature: Weynand et al. (2003) [12] described 0.7% of inadequate samples with the Papspin^® ^system and Bergeron et al. (2003) [13] found 0.14% with the CytoScreen system (which are technically very similar of the Turbitec^® ^system). These authors also demonstrated the superiority of the quality of LBC in comparison with those of CS. With cautions of small number of our samples and using the norms of the Bethesda system 2001, we also found only 0.9% of inadequate cytology [20-24]. Chhieng et al. (2004) also described a similar rate of 0.81% of unsatisfactory sample following the implementation of Bethesda 2001 [20]. Within a comparative study of Thinprep^®^, Autocyte PREP^® ^and the new manual LBC of Digene (DNACITOLIQ^®^, Digene), Alves et al. (2004) also concluded that in spite of the different methodologies, the 3 methods adequately preserved cellular structure for morphologic evaluation [21]. Nam et al. (2004) comparing Thinprep^® ^and a manual LBC, called MonoPrep2^®^, drew similar conclusions [22] All of these authors concluded that manual LBC are cost-effective and provide an alternative method to the currently automated technique of LBC.

Nevertheless, rare are the studies describing the accuracy, in terms of sensibility and specificity, of these manual LBC and particularly for cytocentifugation methods. In our study, to detect the CIN2+, the efficiency of ours LBC, performed by cytocentrifugations, Papspin^® ^and Turbitec^®^, are quite similar. Indeed, their sensitivity are respectively 82.6 % and 75% (p = ns) and their specificity are respectively 92.6 % and 96.2% (P = ns). The small number of Papspin^® ^LBC in our study is due to the subsequent choice for the Turbitec^® ^method which is more cost-effective. By comparison with published results our LBC efficiency performed by cytocentrifugation seems better than CS. A sensitivity of 68% and the specificity of 79% has been indeed reported for the CS and similarly a sensitivity of 76% and a specificity of 86% was reported by others for the Thinprep^® ^[23-26]. Recently, a sensitivity and a specificity quite similar to ours was also reported for the manual LBC of Digene, respectively 75.3% and 86.4% [27].

One main advantage of LBC is that they allow ancillary techniques such as those used in immunocytochemistry or molecular biology [28-32].

The Easyfix^® ^fixative fluid, used in this study for both LBC, is not yet accredited for the use of HCII. With a Kappa test of 0.89 between DNA/HPV PCR and HCII, and with the comparison with the cytological or histological diagnosis, we can conclude that this liquid is efficient for molecular biology and for the HCII technique. Similar conclusions have recently been reached by Leduc et al.(2004) who compared 250 cervical samples which have been fixated with both Easyfix^® ^and Cervical Sampler liquid^® ^(Digene Corp). They found a Kappa of 0.74 [15]. In our study, the sensitivity of HCII, compared with CIN2+, is 86.4 % and the specificity is 39.4 %. These results are similar to those found by Lee et al. (2004) who used Cervical Sampler liquid^® ^(Digene) and found a sensitivity of 94.2% and a specificity of 52.4%[33]. Howard et al. (2002) had similar results with a 81.8% sensitivity and a 51.5% specificity [34]. In our study, the slight difference of specificities is likely been due to the patients selection with high risk HPV infection. Indeed, 50% (41/81) of these HCII false positives showed a CIN1 at the biopsy. Among the others without histological lesion, only 7/40 had no cytological lesion (WNL).

Actually, it is generally accepted that the HCII test is more sensitive and less specific than cytology to identify Hg or Lg-SIL. However, in the case of ASC-US, a combination of HPV DNA and Papanicolaou smears can certainly increase the sensibility of the cervical cancer screening [35-39]. Likely, a cost-effective LBC associated of HPV DNA will probably have a place to reduce cervical cancer in under-developed countries or small laboratories which cannot invest expensive equipment.

We demonstrated by this study that LBC performed by cytocentrifugation are efficient and also allow the HPV DNA preservation in the Easyfix^® ^as no-buffered alcoholic fixative liquid.

Inexpensive LBC performed by cytocentrifugations can be performed by small laboratories which cannot invest in expensive automated equipments.
